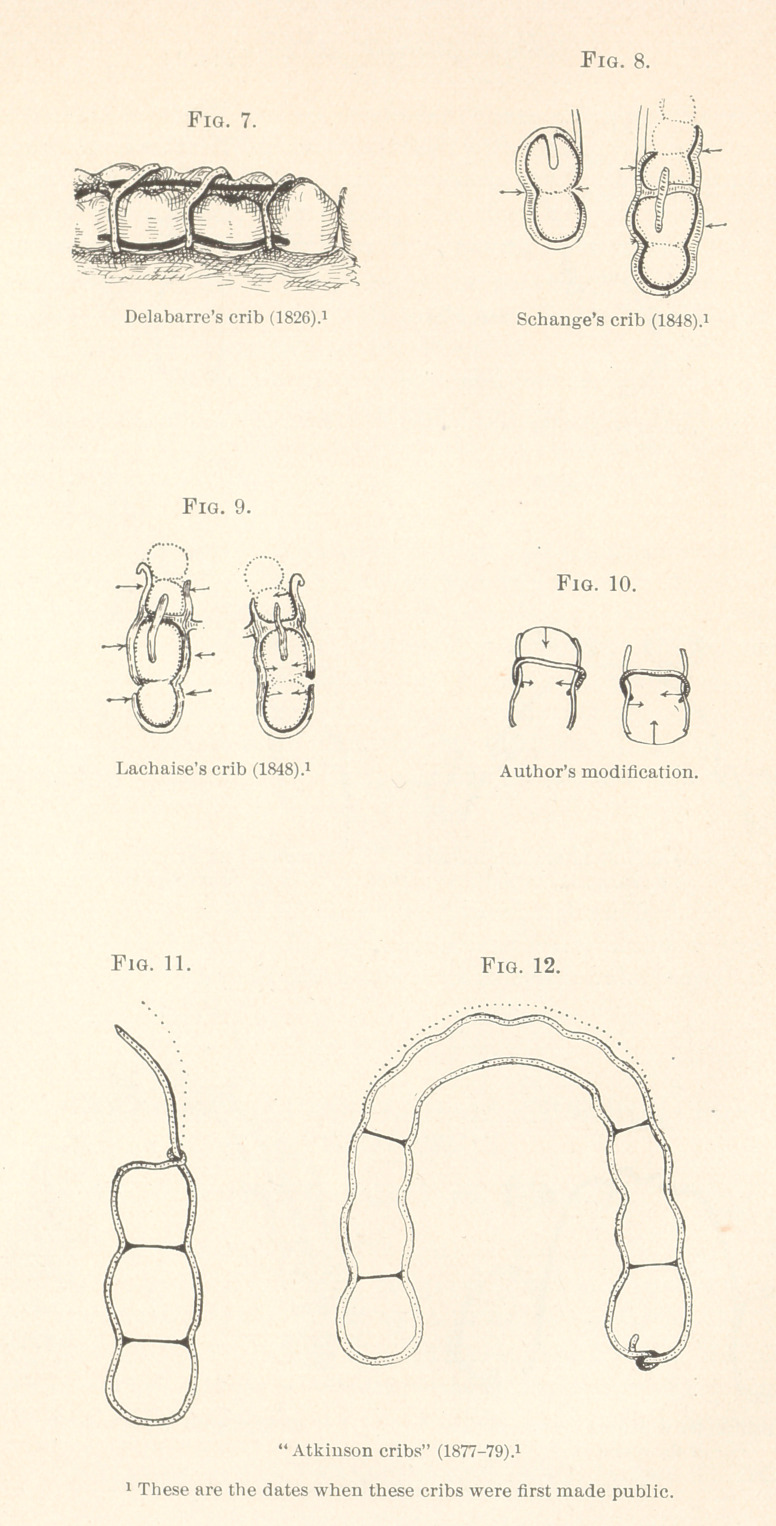# Scallop Wire Mechanism for Regulating Teeth

**Published:** 1894-04

**Authors:** J. N. Farrar

**Affiliations:** New York City


### International Dental Journal.

VOL. XV. APRIL, 1894. No. 4.

### Original Communications.[1]

### SCALLOP WIRE MECHANISMS FOR REGULATING TEETH.

BY J. N. FARRAR, M.D., D.D.S., NEW YORK CITY.

(Continued from page 148.)

IN the March number of this journal was published several *retaining* mechanisms of the scallop wire class that I have found to be of great value in safely *holding teeth in place* after having been regulated. In this paper will be presented several other mechanisms of the same class. These mechanisms, however, are for *correcting irregularities* of the teeth. By these, either a pushing or a pulling force can be caused, but their greater value lies in the pushing qualities. The force may be derived from elastic wire, but the best results are from wire that has little or no elasticity. In these the wire has but few curves; generally two or three, the remainder being plain. The pushing force of the wire is increased by opening wider the curves with broad round-beak pincers, or by an effort to partly straighten the wire. The drawing force is increased by pinching the curves closer.

For anchors to these scallop wires, cribs made after the old plans of Delabarre, Schange, Lachaise, or that which is called the

[1] The editor and publishers are not responsible for the views of authors of papers published in this department, nor for any claim to novelty, or otherwise, that may be made by them. No papers will be received for this department that have appeared in any other journal published in the country.

Atkinson form are practicable. These, which are represented by Figs. 7 to 12 (inclusive), are, however, not as firm as clamp-bands, which may be used with or without plates. For moving only two or three teeth I prefer to use them without. Only such skeleton mechanisms will be explained in this paper.

Fig. 13 represents a small mechanism for moving an instanding upper central incisor forward. It consists of a clamp-band for anchor, a stiff piece of (pushing) wire, W, and a ferrule, having a loose socket. One extremity of the wire W, bent zigzag, is soldered to the anterior part of the lingual side of the clamp-band; the other (being free) is fitted to the socket linked to the ferrule, cemented on the tooth.

The proper point of bearing on the tooth to be moved depends upon the direction and motion desired. If the tooth is to be moved directly outward, the bearing should be against the middle part of the lingual side of the tooth, but if the tooth is to be turned, the bearing should be to one side of the middle.

To increase the pressure on the tooth, the zigzag curves in the wire are slightly opened, as if in an attempt to straighten them.

Fig. 14 represents a similar mechanism for turning a left upper or a right lower cuspid. This one differs from the last one represented, in that it draws upon the tooth, instead of pushing upon it. The pulling wire is situated on the buccal side of the arch. To increase the force, the curves are pinched closer.

Fig. 15 represents a mechanism for moving forward instanding upper or lower centrals. This consists of two bicuspid gum-guard ferrules, A, A (for anchors), a wire bow, W, and two ferrules, F, F. The extremities of the bow are bent zigzag, and the ends are soldered firmly to the lingual sides of the ferrules.

To apply the mechanism, the anterior part of the bow is first placed against the lingual sides of the instanding centrals, and then the gum-guard ferrules are forced on the second bicuspids. To hold the bow in place, it is lodged in open rings soldered to the gum-edge of the lingual sides of the ferrules. These ferrules are cemented on the incisors with phosphate of zinc.

Fig. 16 represents a similar mechanism for moving forward four instanding upper or lower incisors. The difference between this and the last one described lies mainly in the anchors. Instead of gum-guard ferrules being used, these are the author's (slight) modifications of the Delabarre and Schange cribs. My improvement in the crib is confined to uniting the anterior ends of the round side wires by *very thin* gold *ribbons* of rolled wire. These will easily

Fig. 7.

Delabarre's crib (1826).[1]

Fig. 8.

Schange's crib (1848).[1]

Fig. 9.

Lachaise's crib (1848).[1]

Fig. 10.

Author's modification.

Fig. 11.

Fig. 12.

"Atkinson cribs" (1877–79).[1]

slide between the teeth, so as to bear squarely against the anterior approximal sides of the anterior teeth within the cribs, and prevent them (cribs) from being forced posteriorly when the bow is applied behind the incisors. This bow, like that last described, is held against the centrals by ferrules, F, F, having open rings. The force is increased by partly straightening the curves in the wire.

Fig. 17 represents a similarly made mechanism for widening the cuspid part of the upper, or the lower arch. It consists of two clamp-bands, for anchors, a wire bow, and two ferrules having open rings. Like the other mechanisms, the force upon these teeth is increased by straightening, so to speak, the zigzag part of the bow. This bow is also held in place on the cuspids by the ferrules (cemented).

Fig. 18 represents an operation for enlarging the anterior part of the upper arch. The wire bow was held in place by plain ferrules (having open rings) cemented on the centrals and cuspids, and by gum-guard anchor ferrules (not cemented) on the second bicuspids. To increase the forward force against the six front teeth, the posterior parts of the zigzag wire were partly straightened. The lateral force upon the side teeth was increased partly by straightening the curve in the middle of the bow, and partly by the elastic wires on the buccal sides of the anchor-bands. All these various directions of force upon the teeth are indicated by arrows.

This mechanism requires skill to properly form the wire, and properly apply it, but when it is so formed and applied, its actions are very satisfactory. No bunglers can properly make or use successfully this or any of the other mechanisms herein represented.

## Figures and Tables

**Fig. 7. Fig. 8. Fig. 9. Fig. 10. Fig. 11. Fig. 12. f1:**